# Leveraging Smart Health Technology to Empower Patients and Family Caregivers in Managing Cancer Pain: Protocol for a Feasibility Study

**DOI:** 10.2196/16178

**Published:** 2019-12-09

**Authors:** Virginia LeBaron, James Hayes, Kate Gordon, Ridwan Alam, Nutta Homdee, Yudel Martinez, Emmanuel Ogunjirin, Tanya Thomas, Randy Jones, Leslie Blackhall, John Lach

**Affiliations:** 1 University of Virginia School of Nursing Charlottesville, VA United States; 2 University of Virginia School of Engineering & Applied Science Charlottesville, VA United States; 3 Virginia Commonwealth University Health Richmond, VA United States; 4 University of Virginia School of Medicine Charlottesville, VA United States; 5 The George Washington University School of Engineering & Applied Science Washington, DC United States

**Keywords:** cancer, pain, sensors, caregivers, smart health, opioids, palliative care

## Abstract

**Background:**

An estimated 60%-90% of patients with cancer experience moderate to severe pain. Poorly managed cancer pain negatively affects the quality of life for both patients and their family caregivers and can be a particularly challenging symptom to manage at home. Mobile and wireless technology (“Smart Health”) has significant potential to support patients with cancer and their family caregivers and empower them to safely and effectively manage cancer pain.

**Objective:**

This study will deploy a package of sensing technologies, known as Behavioral and Environmental Sensing and Intervention for Cancer (BESI-C), and evaluate its feasibility and acceptability among patients with cancer-family caregiver dyads. Our primary aims are to explore the ability of BESI-C to reliably measure and describe variables relevant to cancer pain in the home setting and to better understand the dyadic effect of pain between patients and family caregivers. A secondary objective is to explore how to best share collected data among key stakeholders (patients, caregivers, and health care providers).

**Methods:**

This descriptive two-year pilot study will include dyads of patients with advanced cancer and their primary family caregivers recruited from an academic medical center outpatient palliative care clinic. Physiological (eg, heart rate, activity) and room-level environmental variables (ambient temperature, humidity, barometric pressure, light, and noise) will be continuously monitored and collected. Behavioral and experiential variables will be actively collected when the caregiver or patient interacts with the custom BESI-C app on their respective smart watch to mark and describe pain events and answer brief, daily ecological momentary assessment surveys. Preliminary analysis will explore the ability of the sensing modalities to infer and detect pain events. Feasibility will be assessed by logistic barriers related to in-home deployment, technical failures related to data capture and fidelity, smart watch wearability issues, and patient recruitment and attrition rates. Acceptability will be measured by dyad perceptions and receptivity to BESI-C through a brief, structured interview and surveys conducted at deployment completion. We will also review summaries of dyad data with participants and health care providers to seek their input regarding data display and content.

**Results:**

Recruitment began in July 2019 and is in progress. We anticipate the preliminary results to be available by summer 2021.

**Conclusions:**

BESI-C has significant potential to monitor and predict pain while concurrently enhancing communication, self-efficacy, safety, and quality of life for patients and family caregivers coping with serious illness such as cancer. This exploratory research offers a novel approach to deliver personalized symptom management strategies, improve patient and caregiver outcomes, and reduce disparities in access to pain management and palliative care services.

**International Registered Report Identifier (IRRID):**

DERR1-10.2196/16178

## Introduction

### Background and Significance

Pain remains a significant problem in cancer care. The biggest fear of patients diagnosed with advanced cancer is not always dying—it is dying in pain [1]. Likewise, family caregivers do not necessarily fear a loved one dying—they fear watching them suffer [2,3]. Unfortunately, for over 15 million Americans coping with cancer [4], these fears are justified. Despite 30 years of effort and imperatives issued by leading health organizations (including landmark reports from the World Health Organization [5], the National Academies of Medicine [6,7], the American Society of Clinical Oncology [8,9], and The National Institutes of Health [10]) to improve pain management, an estimated 60%-90% of patients with cancer still experience moderate to severe pain [11,12]. Poorly managed cancer pain has serious ramifications, negatively affecting sleep, adherence to treatment, mood, and overall quality of life—for *both* patients and their family caregivers [2,11,13,14]. Difficult pain that is not addressed effectively and promptly can escalate, increasing distress and suffering for both patients and caregivers. Witnessing untreated pain is also a significant stressor for family caregivers and can have a lasting, damaging psychological impact [2,15,16]. An especially difficult type of cancer pain to manage is “breakthrough” pain—sudden, often unpredictable, increases in pain [17].

Managing pain in the home context can be extremely challenging. Most cancer symptom management occurs in the ambulatory (outpatient and home) setting, and when patients with cancer are weakened by the effects of treatment or progression of disease, family caregivers commonly assume primary responsibility for managing complex symptoms [3,13,18,19]. For example, family caregivers must be able to detect and interpret physiological, social, and emotional cues to help determine the degree of pain the patient is experiencing, make independent decisions about when and how to intervene, and accurately evaluate and relay to health care providers how well the intervention worked [15]. Complicating cancer pain management is the reality that opioids, a key class of medications used to control serious cancer pain, are potentially drugs of misuse [20]. Given concerns regarding the national “opioid epidemic” [21], it is imperative that patients with cancer and their family caregivers have the support and tools to safely assess and manage pain [22]. We also know that there is a reciprocal dimension to patient and caregiver distress [23-25], but a better understanding of these dyadic and contextual factors are needed to inform effective interventions [2]. Mobile and wireless technology (“Smart Health”) can improve health outcomes for patients with a myriad of health conditions, including cancer [26,27]. A key benefit of leveraging Smart Health technology is the ability to collect a wide range of relevant data passively, minimizing invasiveness and burden—an important consideration for people coping with the stressors of advanced illness. Harnessing Smart Health technology is a critical next step in future research to optimally and comprehensively support patients and family caregivers [28] and has significant potential to help those coping with advanced disease, such as patients receiving palliative care or hospice services [29-31]. To date, technology-based interventions for patients with cancer have largely focused on recording and tracking self-reported symptom data and communicating the results to health care providers [27,32-34]. Research gaps and opportunities include leveraging technology to support family caregivers in managing distressing symptoms, especially pain [35], in the home environment [27,32,36]; identifying patient and caregiver needs in real time [37]; and using theory to inform interventions [28].

### Preliminary Work

**Figure 1:**
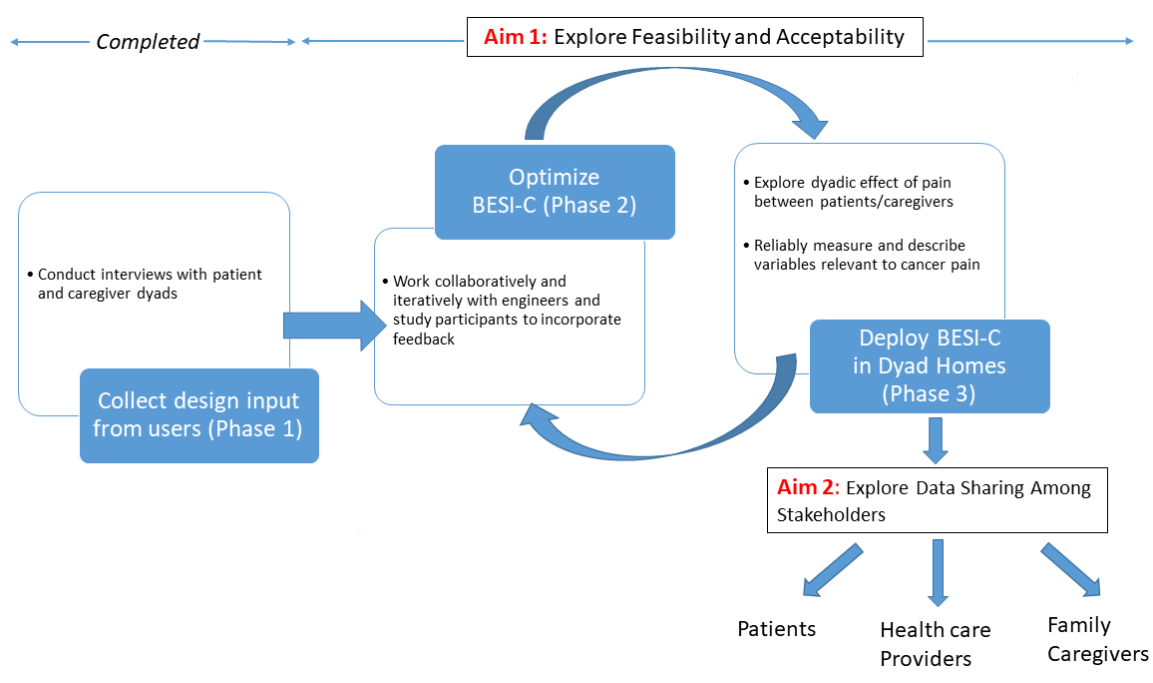
Overview of BESI-C study design. BESI-C: Behavioral and Environmental Sensing and Intervention for Cancer.

This research builds upon pioneering in-home sensing technology originally developed by members of our team to support the care of patients with dementia, known as BESI (Behavioral and Environmental Sensing and Intervention) [38-40]. Simply stated, BESI is a package of sensing technologies set up in a patient’s home, designed to unobtrusively and reliably collect behavioral, physiological, and environmental data. All the data collected by BESI are centrally integrated to paint an in-depth picture about the health and behavior status of individuals and dyads. BESI-C (BESI-Cancer) specifically focuses on factors that may influence cancer pain. Importantly, the design of BESI-C has been informed by end-user feedback gathered during qualitative interviews with cancer patient-family caregiver dyads (Phase 1, Figure 1) recruited from The University of Virginia Palliative Care Clinic. These interviews confirmed that dyads are open to novel technology to help manage cancer pain at home (consistent with prior research [31]), are receptive to pilot testing BESI-C, confirmed our proposed variables to measure with BESI-C, and did not identify additional variables to measure. This preliminary dyad input assisted in the iterative design of BESI-C, particularly related to the smart watch app.

### Theoretical Frameworks

**Figure 2:**
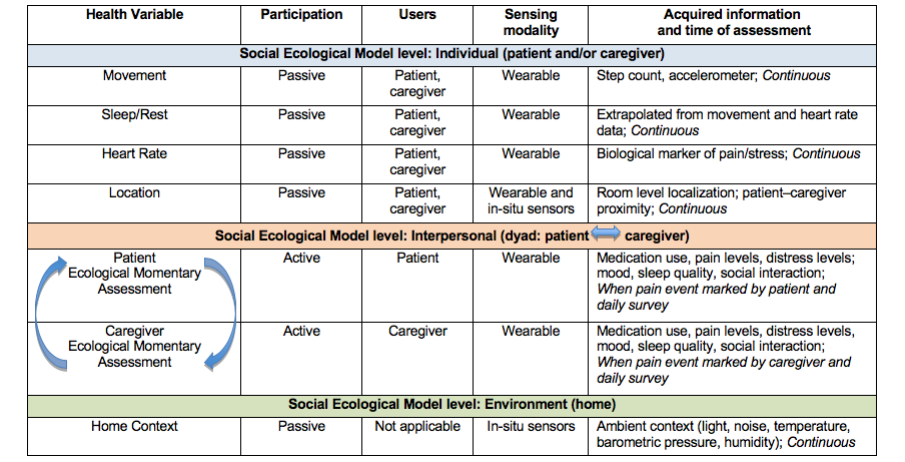
Health variables measured by Behavioral and Environmental Sensing and Intervention for Cancer and related sensing modalities.

This research is grounded in two interrelated conceptual models: the Social-Ecological Model (SEM) and the Dyadic Stress Model. The SEM [41] supports a primary goal of this research, which is to better understand the complex interplay of patient, patient-caregiver dyad, and home environment characteristics that influence the experience of cancer pain. For example, understanding a patient’s individual activity and pain levels (intrapersonal level) will involve consideration of dyadic dynamics that exist between the patient and the family caregiver (interpersonal level) that are, in turn, nested within the broader context of the home setting (environmental levels). Levels of the SEM and how they map to relevant variables measured by BESI-C are summarized in Figure 2. The Dyadic Stress Model posits that life stressors, such as cancer, have a reciprocal impact on patients and their caregivers [42]. For example, understanding how patients’ pain may affect caregiver sleep, and vice versa, is a key aspect of this research.

### Purpose

The overall purpose of this research is to deploy BESI-C and evaluate its feasibility and acceptability among patients with cancer-family caregiver dyads. Our primary aims are to explore the ability of BESI-C to reliably measure and describe variables relevant to cancer pain in the home setting and to better understand the dyadic effect of pain between patients and family caregivers. The secondary aim is to explore how to best share collected data among key stakeholders (patients, caregivers, and health care providers). We hypothesize that patient-caregiver dyads will accept BESI-C in their homes and that BESI-C can reliably capture relevant variables related to cancer-related pain. We are particularly interested in assessing the ability of BESI-C to capture and identify precursors to breakthrough cancer pain, which is notoriously difficult to predict and manage [17]. The ultimate, long-range goal is that data collected from BESI-C can inform and train personalized models that find correlations between environmental contexts and behavioral events, identify precursor patterns related to cancer pain, and provide real-time notifications for early intervention. Early, real-time notifications are important to prevent escalation of pain and distress levels. Currently, the BESI-C system is being utilized in the research domain only, as we must first validate accurate predictive models for breakthrough cancer pain. Once these models have been validated, which will require further work with a larger sample, we hope that BESI-C will be integrated into routine outpatient oncology/palliative care. In this context, BESI-C could deliver real-time personalized interventions to patients and caregivers and share data in real time with patients, caregivers, and health care providers to help direct care management and improve patient and caregiver outcomes.

## Methods

### Recruitment

The study sample is designed to best capture patients and family caregivers coping with difficult cancer pain in the home context. We aim to recruit 20 dyads (patients with cancer and their primary family [informal, unpaid] caregiver) from an academic medical center outpatient palliative care clinic. The number of dyads reflects the scope of this pilot and the primary goal of evaluating feasibility and acceptability. Key patient inclusion criteria include (1) diagnosis of locally advanced or metastatic malignancy, (2) currently taking prescribed opioids for cancer-related pain, (3) ability to understand English and interact with the smart watch, and (4) scores of ≥6 on the NIH PROMIS Cancer Pain Interference scale measures [43]. Key caregiver inclusion criteria include living with the patient full time, identifying as the primary family home caregiver, and the ability to understand English and interact with the smart watch.

### Study Design

**Figure 3:**
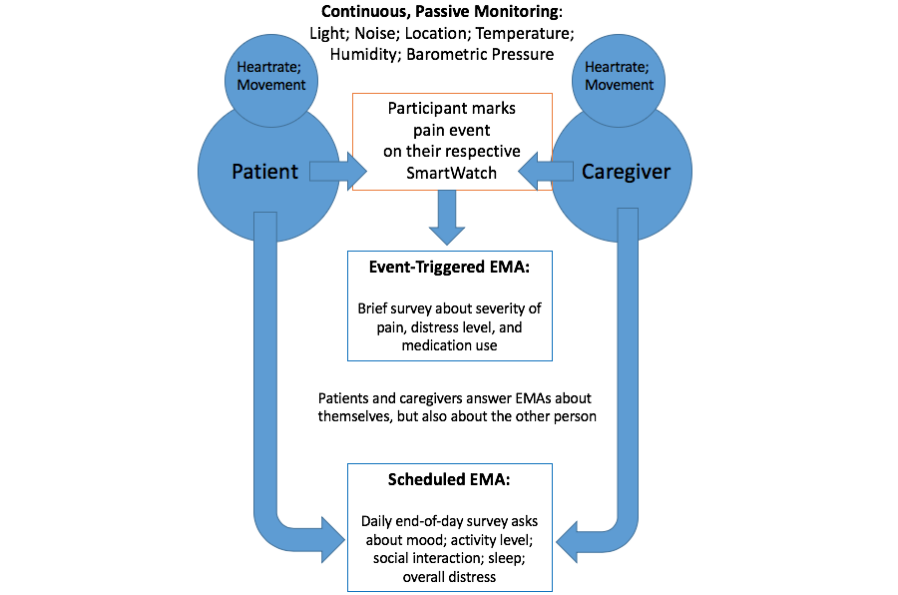
The Behavioral and Environmental Sensing and Intervention for Cancer assessment model. EMA: ecological momentary assessment.

This is a multiphase, descriptive pilot study (Figures 2 and 3). The study was approved by the University of Virginia Institutional Review Board.

### Rationale for Selection of Variables

Variables for data collection via BESI-C (Figure 2) have been selected based on (1) their relevance to pain as identified in the extant literature (eg, fatigue/sleep) [44-46]; (2) technology capabilities of the BESI system [40]; (3) literature documenting the impact of ambient factors such as light, noise, and temperature on the quality of life for patients with advanced illness [47]; (4) attention to reducing study burden in an already stressed and extremely ill patient population [48]; and (5) validation through previously conducted dyad interviews by our research team. Our goal is to collect a range of data variables, grounded in empirical science, while balancing dyad burden, to thoroughly understand contextual and environmental factors that influence pain in this vulnerable group and ultimately develop accurate predictive models, which is a key clinical and scientific gap.

### Architecture of the Behavioral and Environmental Sensing and Intervention for Cancer System

**Figure 4:**
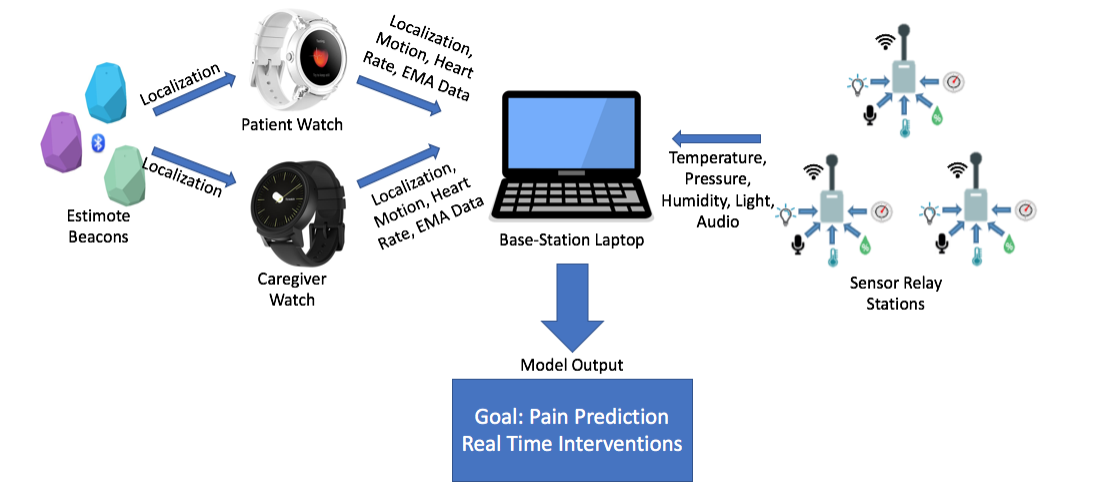
The Behavioral and Environmental Sensing and Intervention for Cancer system architecture for passive data collection: (left to right) Bluetooth Estimote beacons, patient and caregiver smart watches, base station, and sensor relay stations. EMA: ecological momentary assessment.

**Figure 5:**
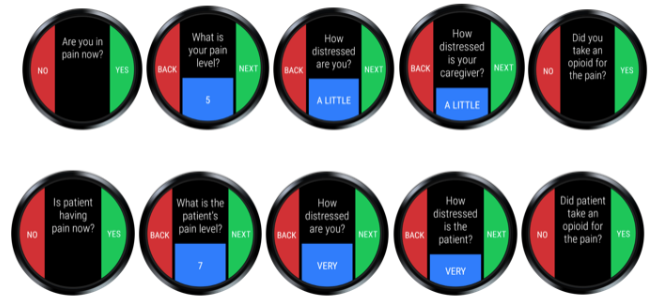
The Behavioral and Environmental Sensing and Intervention for Cancer system architecture for active data collection, examples of smart watch ecological momentary assessments for patient pain events (top), and caregiver pain events (bottom). See Multimedia Appendix 1 for more details.

The in-home BESI-C system includes four primary components (Figures 4 and 5):

#### Smart Watches

Smart watches (Wear OS Fossil Sport Watch, Fossil, Richardson, Texas) will be worn by both the patient and the family caregiver to passively collect photoplethysmogram heart rate and motion data (via accelerometer and step count) and actively collect ecological momentary assessment (EMA) data. Consistent with the scope and aims of this pilot study, we elected to use a well-known commercial off-the-shelf wearable. Although this device does not have 510k clearance, we prioritized wearability of the smart watch with the acknowledgement we are *not* using collected data to direct or alter clinical care [49].

EMAs are brief, contextual assessments commonly used in mobile health to measure symptoms in real-time and send reminders or targeted messages to participants [50]. Each smart watch is programmed with a custom BESI-C app designed for either the caregiver or patient. The BESI-C custom smart watch app includes a platform for patients and caregivers to independently mark and evaluate experienced/perceived pain events, record opioid medication use, and complete a daily EMA survey that asks a series of brief “1-click” questions to evaluate factors such as mood, sleep quality, activity level, and amount of social interaction. Iterative design of the BESI-C custom app has prioritized ease of user interface, speed and simplicity in completion of EMAs, battery life optimization, and low burden and interference with activities such as sleep. EMA survey question format and sequence were developed in consultation with the home institution’s Center for Survey Research. Dyads are asked to wear the watches as much as possible during the deployment and are provided with two watches to swap out when battery life decreases.

#### Sensor Relay Stations

Custom-built environmental sensor stations are strategically deployed in each primary room of the dyad home to passively and continuously collect data on room-level temperature, light, humidity, barometric pressure, and ambient noise. “Primary” rooms include those where participants tend to spend the most time and generally are the living room, bedrooms, and kitchen. We place sensors in consultation with dyads and only with their permission. Environmental data streams are integrated and transmitted to the base station.

#### Bluetooth Beacons

Commercially available Bluetooth Low Energy Estimote Beacons (Estimote Inc, New York) that continuously broadcast device identification information are deployed strategically in the dyad’s home, and their broadcast signals are received by the smart watches. Using the smart watches' received signal strength indicator, the BESI-C app is able to determine the wearer's approximate distance from each beacon, thereby enabling room-level localization of the wearer and an estimation of patient-caregiver proximity.

#### Base Station

A BESI-C configured laptop is placed in an unobtrusive location within the dyad’s home to provide a cyber-physical platform for data offloading and remote system monitoring. Of note, internet access allows remote system monitoring, but is not required for actual data collection. If patients or caregivers are outside of their home, they can still enter data on the smart watch, which are stored locally on the watch until the participant returns home and is reconnected to the BESI-C network. If a dyad does not have reliable internet access in the home, a mobile hotspot is set up to allow remote system monitoring.

### Data Collection

BESI-C is currently being deployed within the homes of patient-caregiver dyads for approximately 10-14 days, consistent with a pilot study and to minimize dyad burden. Investigators remotely monitor BESI-C system fidelity, and deidentified data streams are uploaded to an approved secure cloud server. The environmental sensors and localization beacons are installed in the patient’s home and are not relocated (for example, if the patient is admitted to the hospital), as we are interested in capturing the home context and how that may influence pain. However, the wearable sensor (smart watch) will continue to collect data regardless of the participant location. We are able to discern when dyads are at home by looking at the localization data and reviewing a ground truth daily log that we ask participants to keep during deployment. Passively collected physiological and environmental variables (eg, heart rate, ambient noise) are continuously collected without any interaction needed by the patient or caregiver. Actively collected behavioral and experiential data involve the caregiver or patient interacting with their smart watch to mark the time of a pain episode and describe the event (Multimedia Appendix 1). For example, patients or caregivers are asked to push a button or tap the screen on the smart watch when the patient is experiencing an episode of cancer pain, as perceived by the respective participant. If a pain event is marked, this will generate a brief EMA, which will ask the participant to rate the severity of pain on a simple 0 (no pain) to 10 (worst pain imaginable) scale, their distress level, their perceived partner’s distress level, and if any opioid pain medications or other pain-reducing measures (eg, position change, hot/cold pack) were taken. If an opioid medication or other pain-reducing measure is taken, a repeat EMA is automatically deployed to the participant’s smart watch approximately 30 minutes later to see if pain has decreased. If a participant indicates an opioid was *not* taken for a pain event, we ask them to tell us why (eg, not time yet, concerned taking too much medication, side effects, pain not bad enough, out of pills, some other reason). Additionally, a brief end-of-day EMA survey (approximately 10 questions) asks participants to rate their activity, mood, sleep, social interactions, and overall distress levels over the past day and is used to corroborate passively collected data streams. For example, if a patient reports being “very active” in their daily EMA survey, we can corroborate this with passively collected accelerometer, localization, and step count data. Dyads are also asked to keep a simple, ground-truth, daily paper-based log to record any unusual activity or significant events that occur during the deployment.

We have established *a priori* feasibility and acceptability endpoints for BESI-C data collection, both qualitative and quantitative. *Feasibility* measures are operationalized as (1) logistic barriers related to in-home deployment (eg, physical constraints within the dyad home related to placing environmental sensors); (2) technical failures related to data capture and fidelity (eg, environmental sensors disengaging from the BESI-C network or heart rate data not correlating with accelerometer data); (3) smart watch wearability metrics (Textbox 1); and (4) patient recruitment and attrition rates. *Acceptability* is operationalized as dyad perceptions and receptivity to BESI-C. This endpoint is assessed at the time of removal of BESI-C from a dyad’s home by (1) a brief, structured interview asking about their general experiences with the system and (2) completion of a brief Likert-style survey, which asks participants to agree or disagree with statements such as, “I found the BESI-C system bothersome” or “I would be willing to have BESI-C in my home for a longer period of time.” At deployment completion, we also verify with the dyad any notable clinical or contextual events that may have occurred during the deployment and affected collected data, such as unanticipated visits to the emergency department or prolonged power outages.

Sharing data among key stakeholders is a critical element of understanding how to best support patients and caregivers in managing cancer pain in the home setting. This objective is grounded in principles of learning health systems, which have been advocated by the National Academies and the American Society of Clinical Oncology as an effective strategy to achieve timely, cost-effective, sustainable, targeted, and scalable improvement in health care delivery [51-53]. At the time of removal of BESI-C from a dyad home, we share with patients and caregivers a brief, graphical summary of their deployment data. In sharing these results, we record dyad opinions and feedback regarding the summary sheets in an informal talk-aloud conversation (Textbox 2). We also share these summaries with health care provider clinical collaborators to gather their feedback and opinions about their preferred content of data and how they would like the data displayed/presented.

**Smart watch wearability metrics.**

- Proportion of complete versus incomplete ecological momentary assessments
- Percentage of time smart watch is worn
- Average length of time to complete ecological momentary assessments
- Number of interrupted/“snoozed” ecological momentary assessments
- Number of “dismissed” ecological momentary assessments
- Frequency and duration of times watch put in “do not disturb” mode
- Number of “low battery” notifications

**Example questions asked of dyads to assess data summaries.**

- Does this summary capture the information you are most interested in?
- What information do you find most helpful? Least helpful?
- Is there information you would like to see that is not included in this summary?
- Do you think you need to see different information than your partner? If so, can you provide some examples?
- What information, if any, would you like shared with your health care provider?
- Who else would you like to see this information and why?
- Does this summary seem to accurately represent your experience?
- How would you like this information to be shared with you? (eg, paper print out, website, mobile app)

Privacy and security have been carefully considered for this pilot and are addressed in the following ways: (1) the BESI-C system does not record raw audio data, only preprocessed features related to ambient noise characteristics that do not enable reconstruction of conversation content; (2) the system contains no cameras; (3) sensors are only deployed in rooms approved by the participants and never in highly personal areas such as bathrooms; (4) participants can turn off sensors at any time, simply stop wearing the smart watch, or put the smart watch in to a temporary “do not disturb” mode; (5) all data streams are deidentified, contain no patient identifiers, and are labelled only with a study identification number; and (6) all data collection and streaming are performed within the confines of a local, offline Wi-Fi network via a dedicated router and base station laptop, where the laptop is the sole online device and is equipped with multiple stages of security authorization both locally and remotely.

### Data Analysis

#### Data Collected by the Behavioral and Environmental Sensing and Intervention for Cancer System

Exploring initial data validity is an essential aspect of this exploratory research, as we aim to establish which variables are most important to measure and how we can best capture and analyze these data. Full-scale, real-time data analysis is beyond the scope of this pilot. For these important reasons, BESI-C does not currently alter or direct patient care or medication use in any way; participants are carefully counseled to follow standard procedures for notifying their care team if they experience concerns or changes with their health status. However, using principles of signal processing and machine learning [54,55], we will conduct preliminary analysis to explore the ability of the sensing modalities to infer and detect behavioral events and environmental contexts and to examine patterns, relationships, and concordance between actively and passively collected data. For example, to better understand the dyadic effect of pain, we can mine the data to explore if pain events are equally marked by caregivers and patients; how they are respectively characterized in terms of severity and perceived burden; and how this corresponds with medication use, mobility, sleep, heart rate, and home/room environmental data (eg, temperature, light, noise). We anticipate focusing analysis on *severity* of pain events (those marked as ≥5 and with corresponding moderate/high levels of distress) and *frequency* of pain events (increased number of marked pain events in a specified time period, regardless of severity or distress level). Multimedia Appendix 2 lists example analysis questions and hypotheses. Initial exploratory predictive modeling analysis will identify outcome variables for future research.

#### Feasibility and Acceptability Measures

Feasibility measures will be recorded through a structured research audit log, and descriptive statistics will be used to summarize key metrics such as the number of completed daily EMA surveys and results from Likert surveys. Qualitative data from structured interviews with dyads will be recorded and coded using traditional content analysis to assess patterns and themes [56].

## Results

The two-year grant funding has begun, and Institutional Review Board approval was granted in July 2019. Data collection is currently in progress. As of October 2019, four dyads have been enrolled and completed deployments. We expect the results to be published in summer 2021.

## Discussion

### Overview

This pilot study explores an innovative solution to the challenge of managing cancer pain at home by using a low-burden Smart Health system—BESI-C—to support patients with cancer and their family caregivers. If successful, this model will represent a paradigm shift in how we manage symptoms at home, by being able to monitor, predict, and anticipate distressing symptoms, so we can intervene earlier and more effectively with targeted, personalized approaches. This initial research focuses on breakthrough cancer pain, as it has been shown to be a particularly difficult symptom management issue and because managing pain is a foundational goal of palliative care, a specialty that focuses on optimizing the quality of life for patients and caregivers [57]. However, the long-term vision is that the BESI-C system could be customized to monitor and support a variety of in-home pain scenarios, such as patients enrolled in home hospice programs or patients and caregivers managing chronic, nonmalignant, or postoperative pain.

Our interdisciplinary research makes valuable short-term and long-term contributions in both the clinical and scientific arenas. Specifically, (1) palliative care research is challenging, as the symptom burden is high and interventions must be carefully designed. Smart Health technologies such as BESI-C can collect a wide range of relevant data passively, minimizing invasiveness and burden, which is a critical consideration for this population [48]. (2) Most Smart Health interventions rely on apps that live on people’s smartphones [58-60]. BESI-C is unique in that it is a packaged sensing system that lives in people’s homes through embedded sensors and can collect rich, in-depth data that facilitate personalized system learning, predictive models, and effective targeted interventions [38-40]. (3) How interactions between patients and family caregivers impact symptoms is understudied [28,36], and our research will integrate passively (eg, heart rate) and actively (eg, self-reported pain levels) collected data from *both* the patient and caregiver to better understand how pain may impact the dyadic relationship and vice versa. (4) We know little about specific patient-caregiver behavioral and contextual variables that influence pain and how best to monitor them [61]; as such, BESI-C offers an innovative approach to identify the variables required to develop and deliver timely, tailored, personalized interventions. (5) Managing patient symptoms remotely can be challenging; BESI-C can support patients by providing health care providers with data to inform care management decisions. (6) Pharmacological management of serious cancer pain hinges primarily on opioid therapy, which can be highly effective, but also problematic; our research offers a strategy for safer monitoring and use of opioids in the home setting, which is a particularly critical public health issue [20].

We see tremendous opportunities to advance the work of BESI-C beyond this initial pilot research. Future planned work with BESI-C includes deploying BESI-C with a larger sample of diverse high-risk, high-need populations (eg, dyads living in rural areas); conducting full-scale, real-time, retrospective data analysis to develop predictive models related to symptom manifestation and develop and deploy tailored interventions; continuing to refine and iterate BESI-C’s sensing and data capture capabilities, such as with voice-activated technology; linking BESI-C to electronic medical health records and using principles of Learning Health Systems [51-53] to share data among relevant stakeholders (patients, family caregivers, and health care providers) to inform care management decisions in real-time; testing the ability of BESI-C to impact clinically relevant system-level variables, such as hospital admissions for pain or unplanned discharges from home hospice due to uncontrolled symptoms; addressing scalability, specifically regarding streamlining of deployment procedures and automating remote monitoring and data analysis (eg, in the future, we envision a simplified BESI-C system that could be installed by patients/caregivers themselves); and considering the use of BESI-C with other pain populations, such as patients coping with postoperative pain, neurological disorders, or chronic nonmalignant pain.

### Limitations

A primary limitation of this research (but consistent with the scope and intent of a pilot study) is that we cannot provide clinical interventions or notifications, as we must first confirm data fidelity and develop algorithms for real-time data analysis. Thus, our work at this time is descriptive, and patients and caregivers are given standard of care instructions regarding whom to contact and when, for clinically related questions or emergencies.

### Conclusions

Managing difficult pain at home is stressful for patients with cancer and their family caregivers. Leveraging Smart Health technology such as with BESI-C has significant potential to monitor, predict, and anticipate challenging symptoms and enhance communication, self-efficacy, safety, and overall quality of life for patients and family caregivers coping with serious illness. This exploratory research offers a novel approach to deliver personalized symptom management strategies to improve patient and caregiver outcomes and reduce disparities in pain management.

## Appendix

Multimedia Appendix 1 Detailed functional specifications of Behavioral and Environmental Sensing and Intervention for Cancer smart watch app for patients and caregivers.

Multimedia Appendix 2 Examples of correlations to explore with preliminary data analysis of Behavioral and Environmental Sensing and Intervention for Cancer.

Multimedia Appendix 3 Peer-review report from the American Cancer Society.

## Abbreviations

BESI: Behavioral and Environmental Sensing and Intervention
BESI-C: Behavioral and Environmental Sensing and Intervention for Cancer
SEM: Social-Ecological Model
EMA: ecological momentary assessment

## References

[1] Lemay K, Wilson KG, Buenger U, Jarvis V, Fitzgibbon E, Bhimji K, Dobkin PL (2011). Fear of pain in patients with advanced cancer or in patients with chronic noncancer pain. Clin J Pain.

[2] Smyth JA, Dempster M, Warwick I, Wilkinson P, McCorry NK (2018). A Systematic Review of the Patient- and Carer-Related Factors Affecting the Experience of Pain for Advanced Cancer Patients Cared for at Home. J Pain Symptom Manage.

[3] Martín JM, Olano-Lizarraga M, Saracíbar-Razquin M (2016). The experience of family caregivers caring for a terminal patient at home: A research review. Int J Nurs Stud.

[4] (2019). American Cancer Society.

[5] (2019). Web statement on pain management guidance.

[6] (2014). National Academies Press.

[7] (2011). Relieving pain in America: a blueprint for transforming prevention, care, education and research. National Academies Press.

[8] (2016). ASCO.

[9] American Society of Clinical Oncology (2017). The State of Cancer Care in America, 2017: A Report by the American Society of Clinical Oncology. J Oncol Pract.

[10] (2004). National Institutes of Health.

[11] Goodwin PJ, Bruera E, Stockler M (2014). Pain in patients with cancer. J Clin Oncol.

[12] Deandrea S, Montanari M, Moja L, Apolone G (2008). Prevalence of undertreatment in cancer pain. A review of published literature. Ann Oncol.

[13] Ferrell B, Wittenberg E (2017). A review of family caregiving intervention trials in oncology. CA Cancer J Clin.

[14] Stenberg U, Ruland CM, Miaskowski C (2010). Review of the literature on the effects of caring for a patient with cancer. Psychooncology.

[15] Mehta A, Cohen SR, Ezer H, Carnevale FA, Ducharme F (2011). Striving to respond to palliative care patients' pain at home: a puzzle for family caregivers. Oncol Nurs Forum.

[16] Dumont S, Turgeon J, Allard P, Gagnon P, Charbonneau C, Vézina L (2006). Caring for a loved one with advanced cancer: determinants of psychological distress in family caregivers. J Palliat Med.

[17] Tagami K, Okizaki A, Miura T, Watanabe YS, Matsumoto Y, Morita T, Fujimori M, Kinoshita H (2018). Breakthrough Cancer Pain Influences General Activities and Pain Management: A Comparison of Patients with and without Breakthrough Cancer Pain. J Palliat Med.

[18] Mehta A, Chan LS, Cohen SR (2014). Flying blind: sources of distress for family caregivers of palliative cancer patients managing pain at home. J Psychosoc Oncol.

[19] McGuire DB, Grant M, Park J (2012). Palliative care and end of life: the caregiver. Nurs Outlook.

[20] National Academies of Sciences, Engineering, and Medicine (2017). Pain Management and the Opioid Epidemic: Balancing Societal and Individual Benefits and Risks of Prescription Opioid Use.

[21] (2019). CDC: Centers for Disease Control and Prevention.

[22] Paice JA (2018). Cancer pain management and the opioid crisis in America: How to preserve hard-earned gains in improving the quality of cancer pain management. Cancer.

[23] Badr H, Carmack CL, Kashy DA, Cristofanilli M, Revenson TA (2010). Dyadic coping in metastatic breast cancer. Health Psychol.

[24] Badr H, Shen MJ (2014). Pain catastrophizing, pain intensity, and dyadic adjustment influence patient and partner depression in metastatic breast cancer. Clin J Pain.

[25] Shaffer KM, Jacobs JM, Nipp RD, Carr A, Jackson VA, Park ER, Pirl WF, El-Jawahri A, Gallagher ER, Greer JA, Temel JS (2017). Mental and physical health correlates among family caregivers of patients with newly-diagnosed incurable cancer: a hierarchical linear regression analysis. Support Care Cancer.

[26] Marcolino MS, Oliveira JAQ, D'Agostino M, Ribeiro AL, Alkmim MBM, Novillo-Ortiz D (2018). The Impact of mHealth Interventions: Systematic Review of Systematic Reviews. JMIR Mhealth Uhealth.

[27] Slev VN, Mistiaen P, Pasman HRW, Verdonck-de Leeuw Irma M, van Uden-Kraan Cornelia F, Francke AL (2016). Effects of eHealth for patients and informal caregivers confronted with cancer: A meta-review. Int J Med Inform.

[28] (2017). National Institute of Nursing Research, National Institutes of Health.

[29] MacKenzie Greenle M, Morgan B, Sayani S, Meghani SH (2018). Identifying Mobile Apps Targeting Palliative Care Patients and Family Members. J Palliat Med.

[30] Nwosu AC, Quinn C, Samuels J, Mason S, Payne TR (2018). Wearable smartwatch technology to monitor symptoms in advanced illness. BMJ Support Palliat Care.

[31] Theile G, Klaas V, Tröster G, Guckenberger M (2017). mHealth Technologies for Palliative Care Patients at the Interface of In-Patient to Outpatient Care: Protocol of Feasibility Study Aiming to Early Predict Deterioration of Patient's Health Status. JMIR Res Protoc.

[32] Darlow S, Wen K (2015). Development testing of mobile health interventions for cancer patient self-management: A review. Health Informatics J.

[33] Berry DL, Hong F, Halpenny B, Partridge AH, Fann JR, Wolpin S, Lober WB, Bush NE, Parvathaneni U, Back AL, Amtmann D, Ford R (2014). Electronic self-report assessment for cancer and self-care support: results of a multicenter randomized trial. J Clin Oncol.

[34] Kessel KA, Vogel MM, Schmidt-Graf F, Combs SE (2016). Mobile Apps in Oncology: A Survey on Health Care Professionals' Attitude Toward Telemedicine, mHealth, and Oncological Apps. J Med Internet Res.

[35] Northouse LL, Katapodi MC, Song L, Zhang L, Mood DW (2010). Interventions with family caregivers of cancer patients: meta-analysis of randomized trials. CA Cancer J Clin.

[36] Richardson JE, Reid MC (2013). The promises and pitfalls of leveraging mobile health technology for pain care. Pain Med.

[37] Phongtankuel V, Adelman RD, Reid MC (2018). Mobile health technology and home hospice care: promise and pitfalls. Prog Palliat Care.

[38] Alam R, Dugan J, Homdee N, Gandhi N, Ghaemmaghami B, Meda H, Bankole A, Anderson M, Gong J, Smith-Jackson T, Lach J (2017). BESI: Reliable and heterogeneous sensing and intervention for in-home health applications.

[39] Alam R, Gong J, Hanson M, Bankole A, Anderson M, Smith-Jackson T, Lach J (2017). Motion biomarkers for early detection of dementia-related agitation.

[40] Alam R, Anderson M, Bankole A, Lach J (2018). Inferring physical agitation in dementia using smartwatch and sequential behavior models.

[41] McLeroy KR, Bibeau D, Steckler A, Glanz K (1988). An ecological perspective on health promotion programs. Health Educ Q.

[42] Kayser K, Acquati C, Reese JB, Mark K, Wittmann D, Karam E (2018). A systematic review of dyadic studies examining relationship quality in couples facing colorectal cancer together. Psychooncology.

[43] NIH: National Institutes of Health.

[44] Dodd MJ, Miaskowski C, Lee KA (2004). Occurrence of symptom clusters. J Natl Cancer Inst Monogr.

[45] Dodd MJ, Miaskowski C, Paul SM (2001). Symptom clusters and their effect on the functional status of patients with cancer. Oncol Nurs Forum.

[46] Barsevick AM (2007). The concept of symptom cluster. Semin Oncol Nurs.

[47] Sagha Zadeh R, Eshelman P, Setla J, Kennedy L, Hon E, Basara A (2018). Environmental Design for End-of-Life Care: An Integrative Review on Improving the Quality of Life and Managing Symptoms for Patients in Institutional Settings. J Pain Symptom Manage.

[48] Addington-Hall J (2002). Research sensitivities to palliative care patients. Eur J Cancer Care (Engl).

[49] Bove La (2019). Increasing Patient Engagement Through the Use of Wearable Technology. The Journal for Nurse Practitioners.

[50] Shiffman S, Stone AA, Hufford MR (2008). Ecological momentary assessment. Annu Rev Clin Psychol.

[51] Friedman C, Rubin J, Brown J, Buntin M, Corn M, Etheredge L, Gunter C, Musen M, Platt R, Stead W, Sullivan K, Van HD (2015). Toward a science of learning systems: a research agenda for the high-functioning Learning Health System. J Am Med Inform Assoc.

[52] Sledge GW, Hudis CA, Swain SM, Yu PM, Mann JT, Hauser RS, Lichter AS (2013). ASCO's approach to a learning health care system in oncology. J Oncol Pract.

[53] Smith M, Saunders R, Stuckhardt L, McGinnis J (2013). Best Care at Lower Cost: The Path to Continuously Learning Health Care in Americahealth care in America. Institute of Medicine.

[54] Lötsch J, Ultsch A (2018). Machine learning in pain research. Pain.

[55] Nwosu AC, Collins B, Mason S (2018). Big Data analysis to improve care for people living with serious illness: The potential to use new emerging technology in palliative care. Palliat Med.

[56] Krippendorff K (2018). Content Analysis: An introduction to its methodology. 4th edition.

[57] (2018). National Consensus Project for Quality Palliative Care.

[58] Sun Yunheng, Jiang Feng, Gu Juan J, Wang Y Ken, Hua Hongwei, Li Jing, Cheng Zhijun, Liao Zhijun, Huang Qian, Hu Weiwei, Ding Gang (2017). Development and Testing of an Intelligent Pain Management System (IPMS) on Mobile Phones Through a Randomized Trial Among Chinese Cancer Patients: A New Approach in Cancer Pain Management. JMIR Mhealth Uhealth.

[59] Bender JL, Yue RYK, To MJ, Deacken L, Jadad AR (2013). A lot of action, but not in the right direction: systematic review and content analysis of smartphone applications for the prevention, detection, and management of cancer. J Med Internet Res.

[60] Lalloo C, Jibb LA, Rivera J, Agarwal A, Stinson JN (2015). "There's a Pain App for That": Review of Patient-targeted Smartphone Applications for Pain Management. Clin J Pain.

[61] Chi N, Demiris G, Pike KC, Washington K, Oliver DP (2018). Pain Management Concerns From the Hospice Family Caregivers' Perspective. Am J Hosp Palliat Care.

