# Supplementary material for: Leveraging Smart Health Technology to Empower Patients and Family Caregivers in Managing Cancer Pain: Protocol for a Feasibility Study
Source: JMIR Res Protoc. 2019 Dec 9;8(12):e16178. doi: 10.2196/16178 (PMC6928698; doi:10.2196/16178)

Duty Cycle Process

Unless stated otherwise, system sensor duty cycling are as follows:

-Pedometer Sensor (Step Count)
- Always on

-Accelerometer Sensor
- Disabled during sleep mode
- Sampling at ~15Hz

-Photoplethysmography Sensor (Heart Rate Sensor)
- Disabled during sleep mode
- 5 Minute Duty Cycle
    - Enabled for 30 seconds
    - Disabled for 4 minutes and 30 seconds

-Localization Sensor (Beacon/Estimote Sensor)
- Disabled during sleep mode
- 1 Minute 30 Second Duty Cycle
    - Enabled for 15 seconds
    - Disabled for 1 minute and 15 seconds

***** During the Pain and Followup EMA, the Heart Rate Sensor and Localization Beacon are running continuously until end of survey. (No duty Cycling Enabled)
***** If no steps are detected after the Localization sensor has run 5 times, the sleep function is automatically enabled.
***** The sleep function disables all sensor and data gathering except for the Pedometer Sensor.

BESI-C Application Screens

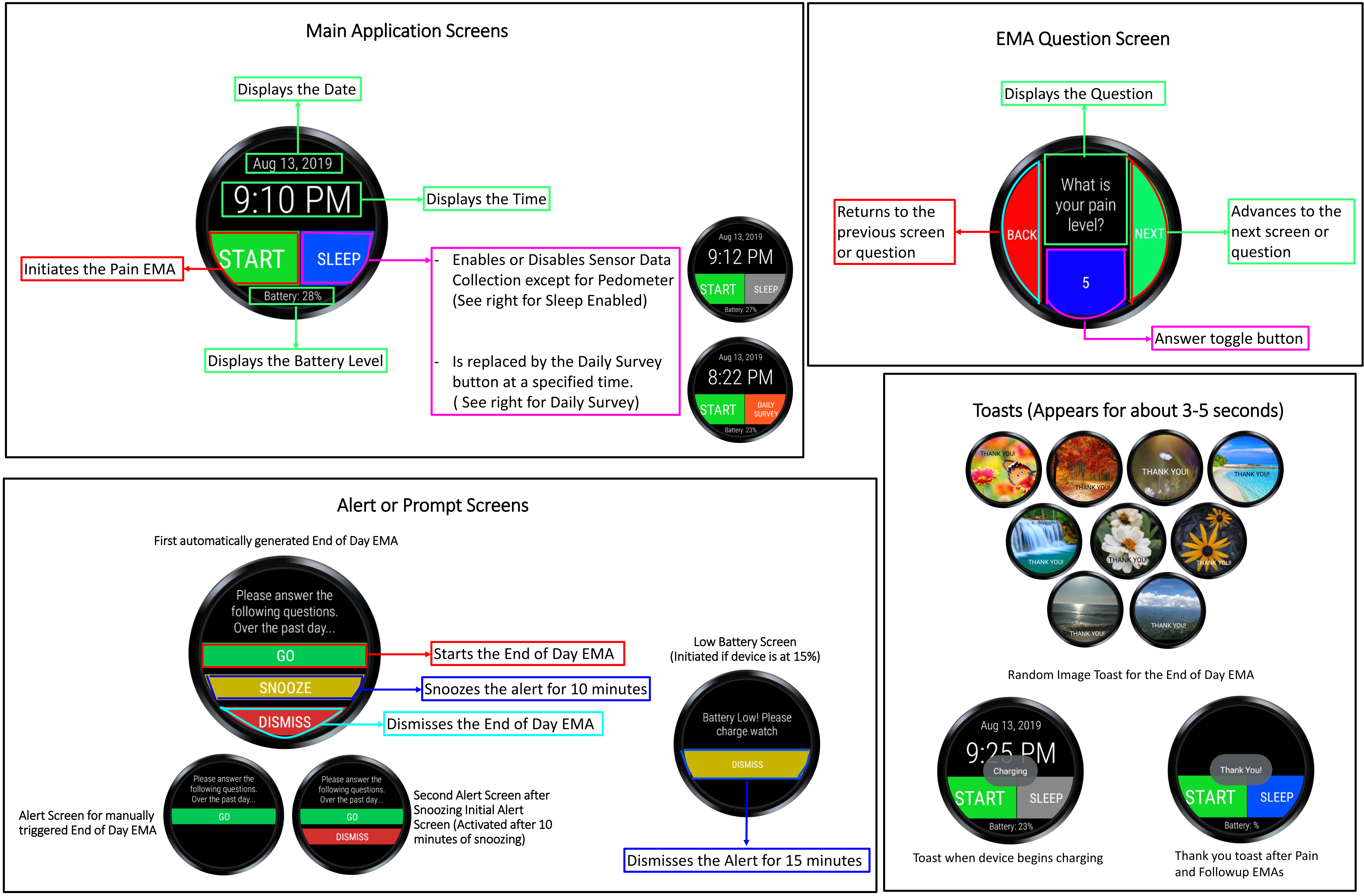

Patient Pain EMA

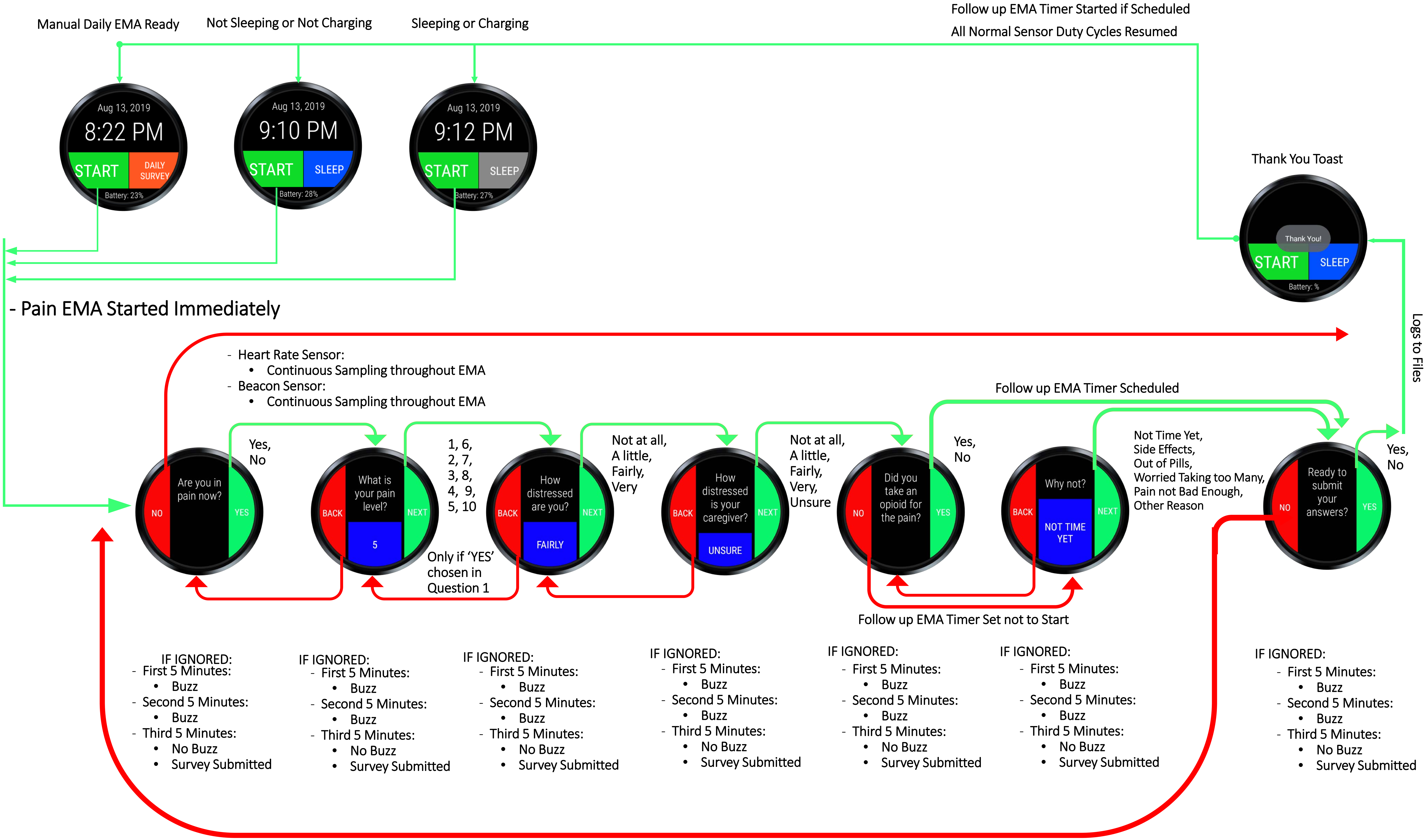

Patient Followup EMA

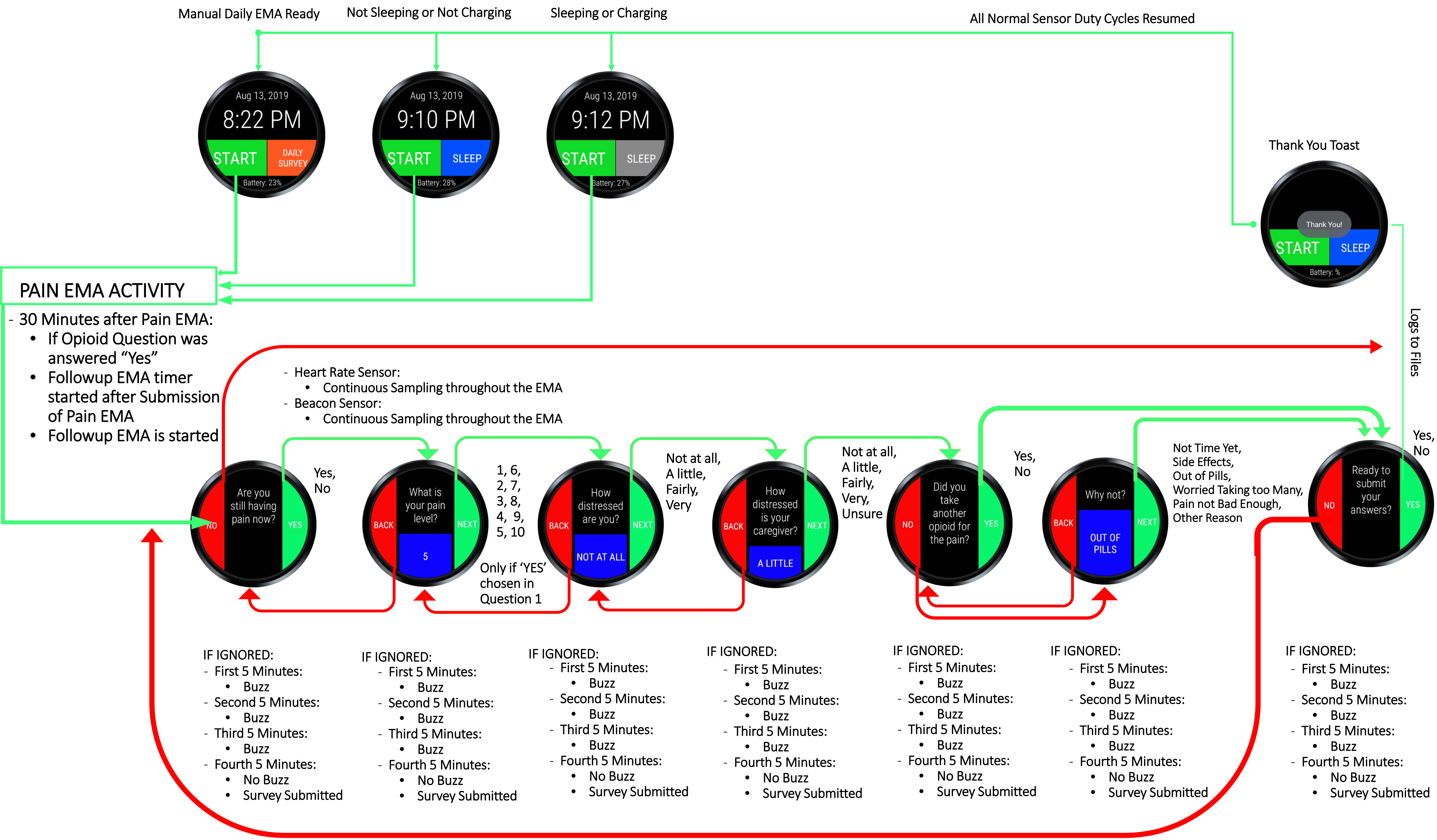

Patient Manual End of Day EMA

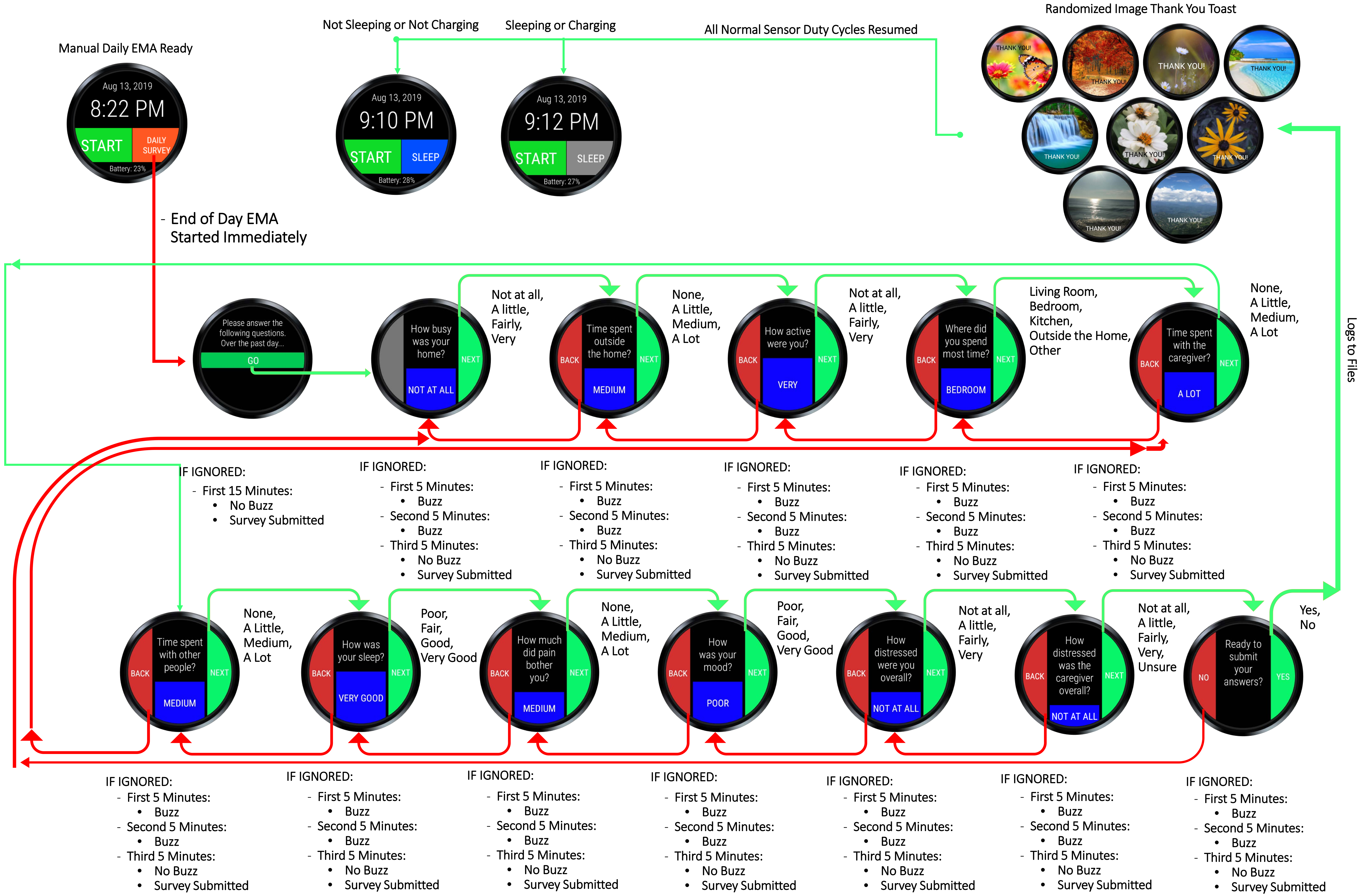

Patient Automatic End of Day EMA

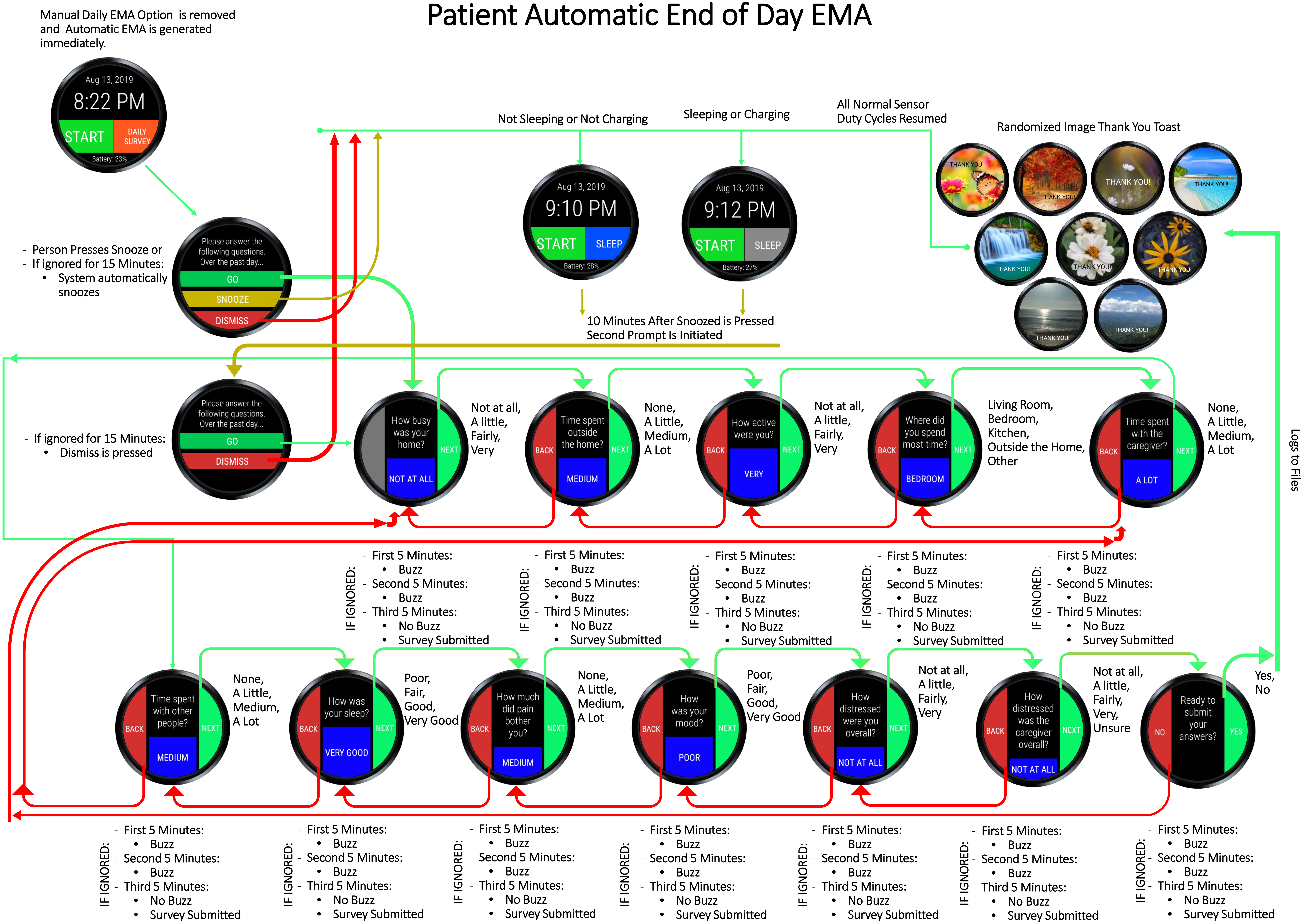

Caregiver Pain EMA

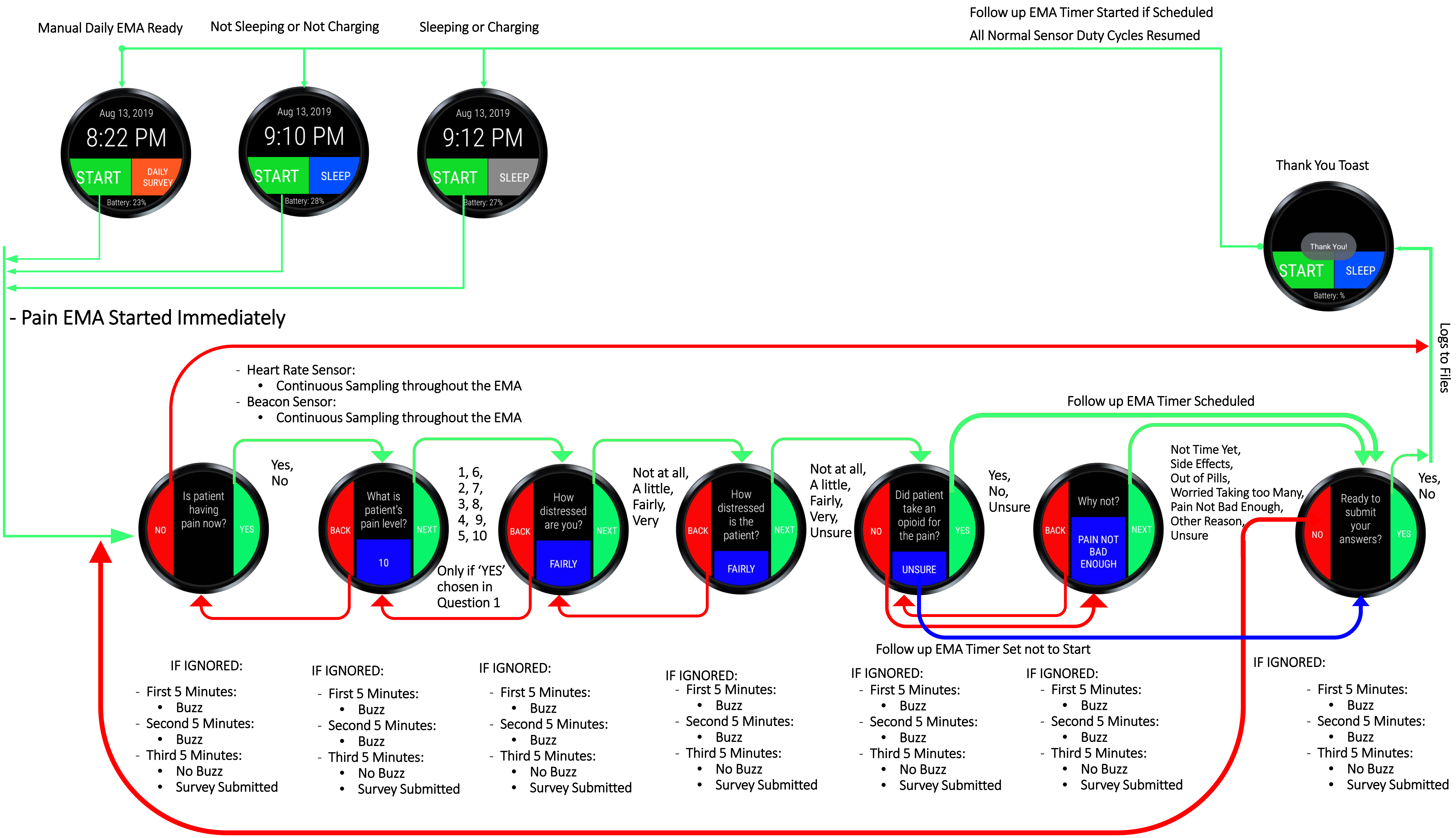

Caregiver Followup EMA

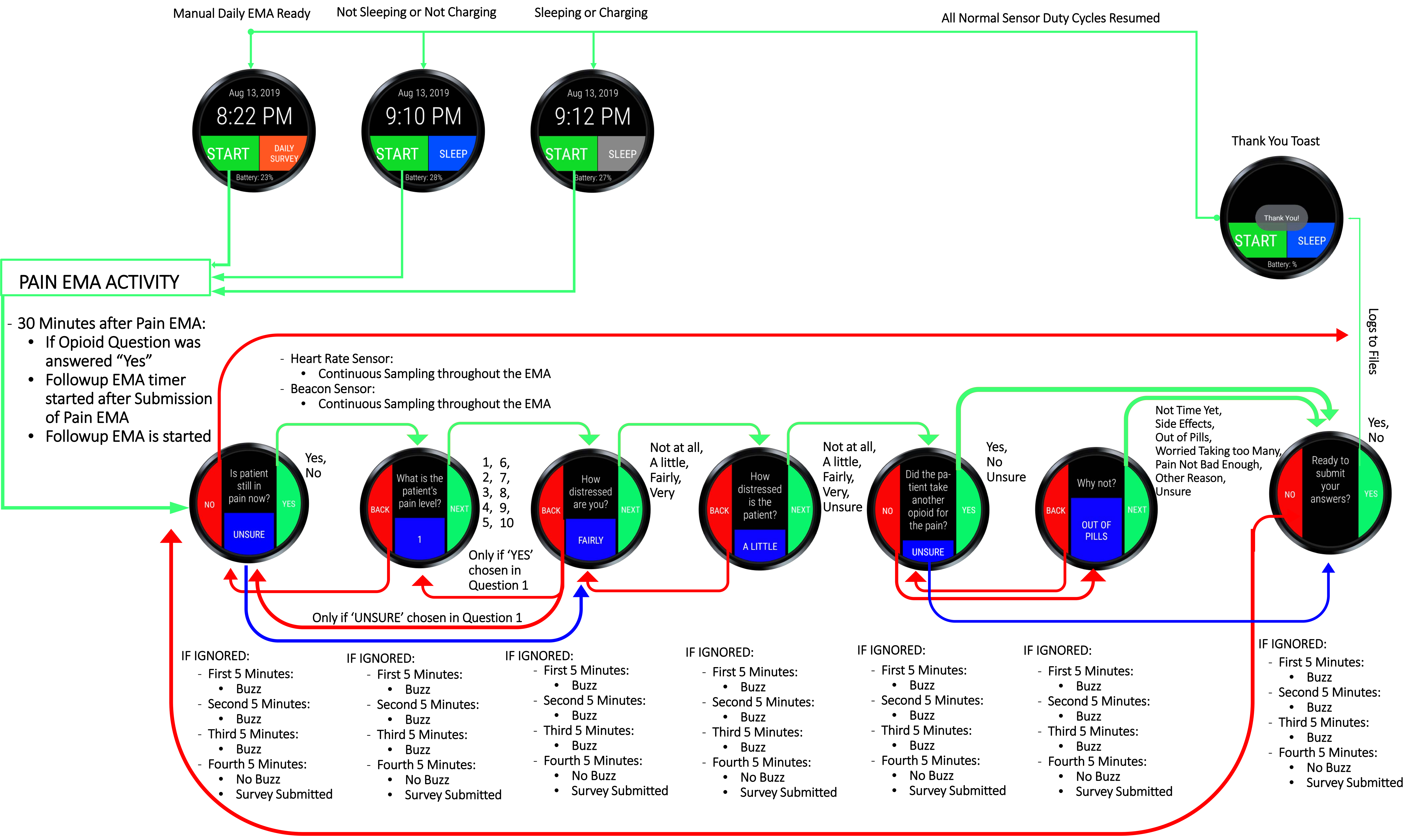

Caregiver Manual End of Day EMA

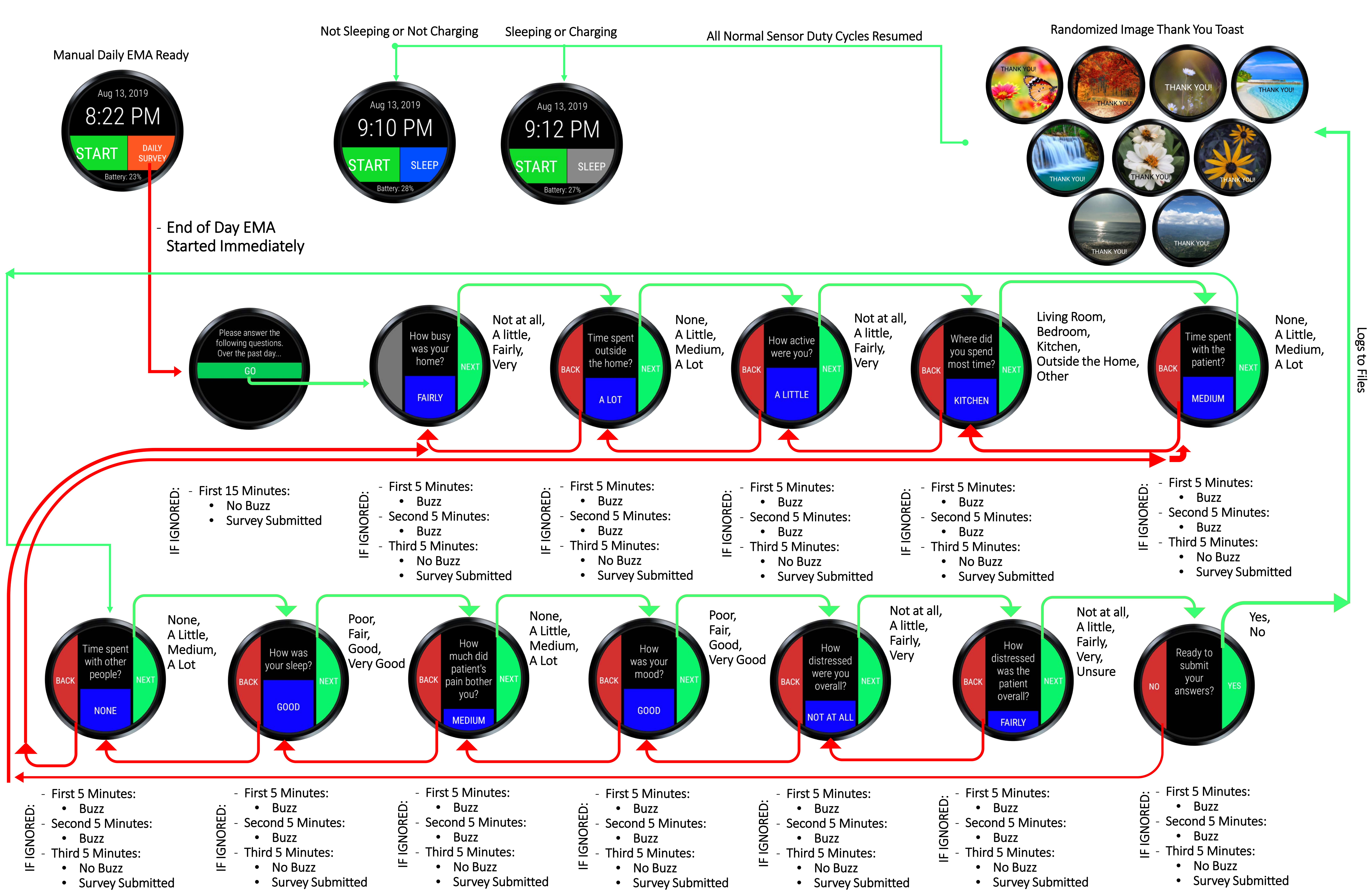

Caregiver Automatic End of Day EMA

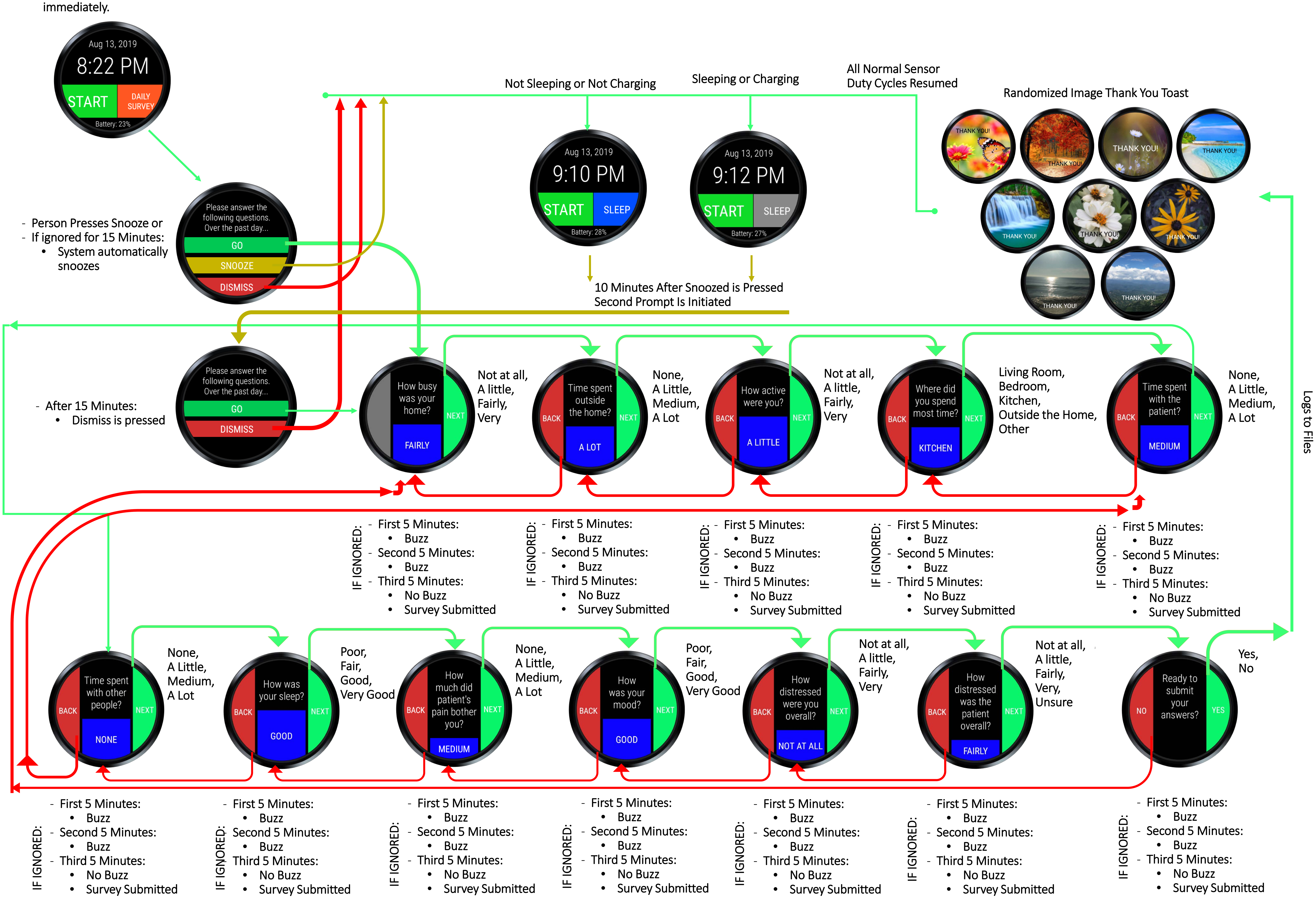

Supplement: Multimedia Appendix 1 [file resprot_v8i12e16178_app1.pdf]
