# Supplementary material for: Leveraging Smart Health Technology to Empower Patients and Family Caregivers in Managing Cancer Pain: Protocol for a Feasibility Study
Source: JMIR Res Protoc. 2019 Dec 9;8(12):e16178. doi: 10.2196/16178 (PMC6928698; doi:10.2196/16178)
Supplement: Multimedia Appendix 2 [file resprot_v8i12e16178_app2.docx]

**Multimedia Appendix 2**

Examples of correlations to explore with preliminary data analysis of BESI-C.

| **Question** | **Variables** | **Hypothesis/Comments** |
| --- | --- | --- |
| What *environmental* factors correlate with frequency of *patient and caregiver marked* pain events, particularly those rated as ≥5 severity? | IV^1^: Room temperature; light; noise; humidity, barometric pressure; room location; patient/caregiver proximity  DV^2^: Number of marked pain events with severity of ≥5 in a 24-hour period | ↑ temperature, light, noise, barometric pressure and humidity will ↑ frequency and severity of pain events; certain room locations and ↑ patient and caregiver proximity will ↓ frequency or severity of pain events |
| What *behavioral/physiological* factors correlate with frequency of *patient-marked* pain events, particularly those rated as >5 severity? | IV: Activity/step count; sleep quantity and quality; HR  DV: Number of marked pain events; pain events rated ≥5 | ↓ sleep quantity and quality will ↑ frequency and severity of pain events; ↑activity/step count = ↑ frequency and severity of pain events |
| What *behavioral/physiological* factors correlate with frequency of *caregiver-marked* pain events particularly those rated as ≥5 severity? | IV: Activity/step count; sleep quantity and quality; HR  DV: Number of marked pain events; pain events rated ≥5 | ↓ sleep quantity and quality will ↑ frequency and severity of pain events; ↑ activity/step count will ↓ frequency and severity of pain events |
| How does physical proximity influence concordance between patient marked pain events and caregiver marked pain events? | IV: Beacon localization of patient/caregiver interaction  DV: Number of matched patient and caregiver marked pain events^3^   *** | ↑ caregiver/patient proximity = ↑ proportion of concordance of marked pain events |
| When there is a matched marked pain event, what is the concordance between patient and caregiver repeat EMA responses? | IV: Matched marked pain event  DV: Patient and caregiver ratings of pain severity; distress; whether they took medication and if they needed additional medication | Particularly interested in how patients perceive/rate caregiver distress and how caregivers perceive/rate patient distress |
| What is the correlation between marked pain events and daily EMA survey responses for both patients and caregivers? | IV: Mood; activity level; social interactions; sleep quality/quantity; overall pain; overall distress  DV: Frequency of marked pain events, severity of pain events, frequency of medication use, distress levels | Patients and caregivers will mark ↑ pain events more frequently and of higher severity with ↑ medication use on days they report ↓ mood, ↓ sleep quality/quantity,  ↓ social interaction, ↑ overall distress levels and ↑ overall pain interference levels |

^1^ IV = independent variable; ^2^DV = dependent variable; ^3^ Matched pain events are those marked independently by patient and caregiver within approximately 15 minutes of each other.
