# Supplementary material for: Leveraging Smart Health Technology to Empower Patients and Family Caregivers in Managing Cancer Pain: Protocol for a Feasibility Study
Source: JMIR Res Protoc. 2019 Dec 9;8(12):e16178. doi: 10.2196/16178 (PMC6928698; doi:10.2196/16178)
Supplement: Multimedia Appendix 3 [file resprot_v8i12e16178_app3.PDF]

March 6, 2019

To: Postdoctoral Fellowship, Research Scholar Grant, Mentored Research Scholar Grant, and Clinician Scientist Development Grant Applicants

From: Elvan C. Daniels, MD, MPH
Scientific Program Director, Cancer Control and Prevention Research

Re: Critiques

Please see the critiques of your application at the end of this memorandum. This is to provide you with additional information concerning the peer review process, and to answer some frequently asked questions.

1. Each application is assigned to at least two committee members, who submit their critiques before they attend the Peer Review Committee meeting. The critiques are made available to all members of the committee before the proposal is discussed. Critiques can be revised by the reviewers in light of discussions at the open meeting. Due to practical time constraints, not all applications are orally discussed during the meeting of the Peer Review Committee.

2. The terms used to describe the proposals are: **Outstanding, Excellent, Good, Fair and Non-Competitive** in decreasing order of enthusiasm. When the proposal is discussed in the open meeting, or in online threaded discussions, the reviewers present their critiques to the entire committee. If the opinions of the primary and secondary reviewers are very different, the committee strives to ensure that all of the important issues are clarified to the full committee prior to voting. This can result in a preliminary review being “talked up” or “talked down” so that the eventual priority score may occasionally seem inconsistent with one of the reviews. Each member of the committee, including stakeholders, scores the proposal, and the final ranking is based on the average of individual scores. We do not provide scores to the applicants because we believe that it is more useful to focus on the overall rating and recommendations of the peer review committee rather than the numeric score.

3. The Council will carry out the final review of the proposal at its meeting in mid-March 2019 and make final recommendation for the paylines for each Peer Review Committee. No additional unsolicited information can be accepted concerning the applications unless requested by the Program Director.

4. We allow one resubmission of Postdoctoral Fellowships and two resubmissions for Research Scholar Grants and for Clinician Scientist Development Grants. The Mentored Research Scholar Grant has been phased out as of fall 2018. Resubmissions for the MRSG will no longer be accepted. In all cases, applicants are urged to consult the ACS policies on eligibility, available at www.cancer.org, before resubmission. Applicants are encouraged to read their critiques carefully and thoughtfully consider the comments and suggestions of the reviewers. Following review of the critiques, applicants are encouraged to schedule time to speak with their Program Director who can provide additional insights about the review of the application by the peer review committee.

The American Cancer Society uses scheduling software that will allow you to directly schedule a conference call with their Program Director during the month of March and April to discuss the critiques of your grant application. This system saves a great deal of time for you and for our staff in arranging the many calls between applicants and the program directors. Using this tool, you will be able to see the available times on my schedule and sign up for an appointment time. In addition, if a change needs to be made, you can log back into the site and adjust the scheduled time on the calendar. If you find that the schedule is full or you are unable to find a suitable time, please contact Chanda Felton (chanda.felton@cancer.org) and she will endeavor to find a time to arrange a call.

If, for any reason, this system does not work properly for you, please email Annette Jordan (annette.jordan@cancer.org) for help with the program.

Instructions:

1. Go to https://www.appointmentquest.com/provider/2080155035
2. Select the Make Appointment for Grant Application Follow-Up box
3. Select Ellie Daniels as the Program Director
4. Check Availability
5. Choose a time and Make Appointment
6. Complete the new Customer Profile
7. Fill in the Committee Code for application cycle *(i.e., CPPB, CPHPS, PCSM)*
8. You will receive an automated email with the time and day chosen for the call

Please note that all information will be purged from the system at the end of April, i.e., no personal information is ever made available to outside organizations.

PILOT AND EXPOLORATORY PROJECT CRITIQUE TEMPLATE

Application ID #:	133047	Reviewer Number (1 or 2)	1
Applicant Name	LeBaron, Virginia		
Project Title	**Using mHealth to support patients and caregivers in managing cancer pain**		

Summary

This second resubmission application aims to address a common yet underemphasized issue in cancer care: poorly-managed pain. It proposes a mobile health intervention called BESI-C (Behavioral and Environmental Sensing Intervention for Cancer) to address cancer pain at home. The primary goal of this study is to test the feasibility and acceptability of deploying BESI-C in the homes of patients and caregivers who are seen in an outpatient oncology palliative care clinic at the University of Virginia. The secondary objective is revised to now explore methods for sharing collected data among relevant stakeholders to inform future interventions to improve the patient/caregiver experience of pain.

The application scored "excellent" on its first resubmission, having addressed major issues related to the definition and measurement plan for "feasibility," and a lack of clarity about mentorship. Some minor critiques remained, regarding next steps in this program of research, how to bridge the gap between just collecting lots of data vs actually making it useful and actionable, and also some concerns about the cost of BESI-C. The applicant aims to address these with this second resubmission. Of note, the team has started some work on this research project, such that the prior first aim involving structured interviews with patient-caregiver dyads has actually already been completed and is now included as preliminary data with this application.

PART I CANDIDATE:

The candidate is a PhD trained nurse scientist, with an Assistant Professor level faculty appointment at UVa, which she has held since July 2015 (after completing post-doctoral training in cancer and health disparities at Dana Farber in Boston). She also held a clinical faculty appointment at the University of Arizona, from 2006 through 2011, and has a history of doing work around cancer pain management and access to palliative care services in India, as part of a Fulbright Fellowship and prior pre-doctoral grant support from the American Cancer Society (2008-12). She has a new R21, assessing the impact of mHealth approaches on cancer pain management and palliative care in Nepal, as well as a local UVa seed pilot grant, and is co-investigator on another UVa cancer control grant. The candidate has a positive track record of disseminating research findings via peer-reviewed publications, related to prior funding she has held. She has published several new papers since the last submission, now with a total of 20 papers, per the Medline bibliography link from her biosketch that has since been corrected. She completed training at the NIH mHealth Institute, of relevance to this proposal, and is being mentored by Randy Jones, PhD, in the School of Nursing, who has again provided a letter of support with this resubmitted application.

PART II RESEARCH PLAN:

- SIGNIFICANCE: This proposal addresses an important area of clinical practice and patient/family experiences of illness: pain management. It also recognizes key next steps in mHealth applications to practice: moving from the collection of more limited, structured symptom-oriented data, to more robust data collection utilizing biosensors and beyond. This revised proposal now seeks to also explore methods for sharing collected data with relevant stakeholders, which is a key gap in current understanding and implementation science regarding patient-generated health data.

- CANCER RELEVANCE: given the prevalence of cancer-related pain, the growing interest in mHealth, and technological advancements made in the last decade regarding biosensors and wireless connectivity, this area or research is highly-relevant.

- INNOVATION/IMPROVEMENT: The use of a non-app-based system to assess patients' experiences of illness in the home is novel, and innovative. Bringing caregivers into the equation adds another important layer to this work, as this area is relatively unexplored in mHealth to date.

- INVESTIGATOR/ RESEARCH TEAM: The proposal builds upon existing resources and strengths of the research team, including the PI's training as an mHealth Institute Scholar, and a co-investigator's experience building the BESI system previously. Since this system does not need to be created from scratch, but rather will be adapted for the patient/caregiver dyad and for those with cancer, the team seems well-positioned for success. Dr. LeBaron is a tenure track faculty member who will be eligible for tenure and promotion in 2020. She initially had support from the Roberts Scholar program (a designation for promising junior faculty that provides 2 years of protected research time) and has leveraged this into R21 and other funding already.

- APPROACH:

  Aim 1 assesses feasibility and acceptability of BESI-C in a cancer patient and caregiver dyad. This Aim appropriately employs explicitly defined measures of feasibility and acceptability, with the former (feasibility) mostly being about study eligibility, enrollment, and completion of required study elements, which is very appropriate and traditional. The latter (acceptability) is mostly based in qualitative assessments and brief satisfaction surveys, which again is very traditional and appropriate for a study like this. Since the prior resubmission, the team has completed initial qualitative interviews with 10 patient/caregiver dyads and are thus well-poised to take this work to the next level with the requisite funding. Participants reported the concepts inherent in BESI-C to be highly acceptable and were interested in participating in a future pilot.

  Revised Aim 2 seeks to explore methods for sharing collected data with key stakeholders, to inform future interventions that improve the patient/caregiver experience of cancer pain. Of note, the applicant has identified an R01 mechanism to leverage these pilot data into further funding and has talked with an NINR Program Officer about the project.

- ENVIRONMENT: The scientific and clinical environments at UVa are excellent, and the connection with the engineering program and Dr. Lach is particular important for this proposal.

PART III BUDGET AND JUSTIFICATION OF BUDGET:

BESI-C is expensive, as noted in prior reviews. This does not limit my enthusiasm for this proposal, and the importance of the work overall, however.

PART IV USE OF HUMAN OR ANIMAL SUBJECTS:

There is appropriate consideration for protection of human subjects. No concerns noted.

PART V OVERALL RECOMMENDATIONS:

The applicant has made additional incremental changes to the approach in response to prior reviews and has successfully completed initial stages of the previously-proposed pilot work. She is thus now poised to start this study, if funding is awarded. The revised aim 2 is nicely responsive to reviewer feedback regarding the utility of data collected via BESI-C, and even incorporates principles of Learning Health Systems, a very relevant and timely framework for this work. I am highly enthusiastic about this proposal, and the importance of this work. At this time, I see no remaining significant concerns with the approach.

PILOT AND EXPOLORATORY PROJECT CRITIQUE TEMPLATE

Application ID #:	133047	Reviewer Number (1 or 2)	2
Applicant Name	LeBaron		
Project Title	**Using mHealth to support patients and caregivers in managing cancer pain**		

PART I CANDIDATE:

Dr. LeBaron is an Assistant Professor in the University of Virginia Schools of Nursing and Medicine. She is a Roberts Scholar and a Full Member of the University of Virginia Cancer Center. She has expertise in caring for patients with advanced cancer, palliative care, cross-cultural factors associated with pain and pain management. She was a Fulbright Fellow and American Cancer Society Pre-Doctoral Scholar and led a 9-month ethnography in South India exploring barriers to pain management and opioid availability in a government cancer hospital. She is currently the Principal Investigator on an R21 to develop an mHealth intervention to implement cancer pain management guidelines in Nepal and participated in the 2018 Smart and Connected Health NSF workshop as an attendee. She has held leadership positions in oncology and is on the Editorial Board for Oncology Nursing Forum and has conducted research internationally in resource-constrained settings focused on nursing and palliative care. There were some concerns raised in her previous submission about lack of publication record and this submission has been corrected to reflect that Dr. LeBaron has had 10 PubMed publications since 2015, 5 of which have occurred since the original submission of this project. Accordingly, her research and clinical experience indicate she is well-poised to undertake the proposed project.

PART II RESEARCH PLAN: *(Reply to previous reviews, if applicable*)

This proposal reflects a second resubmission of this project. Throughout both resubmissions, Dr. LeBaron has thoughtfully attended to reviewer critiques and revised her application accordingly.

The focus of this project, cancer pain, is of high interest and clinical relevance given that cancer pain remains undertreated despite the availability of appropriate pharmacological and non-pharmacological interventions. The field of mHealth is a potential means of addressing cancer pain management in the home setting. The proposed project attempts to identify variables associated with pain experience in the home setting, optimize a home-based pain sensing system tailored for cancer patients, and evaluate the feasibility and acceptability of this mHealth system.

<u>Significance:</u> Pain is a significant problem in patients with cancer and despite available interventions and management guidelines, remains undertreated. Pain management at home is particularly challenging with many barriers to optimal treatment, including patient- and family-related factors. The growing field of mHealth provides an avenue for optimizing pain management in the home setting. Accordingly, the proposed study addresses a significant issue in the context of cancer and proposes a novel means of assessing pain experience at home.

<u>Cancer relevance:</u> the proposed project directly addresses the pain experience of patients with cancer in the home setting. Caregivers are of high importance to include in research on cancer pain management given the significant role that caregivers play in symptom management in the home setting.

<u>Innovation/improvement:</u> Currently, pain at home in cancer patients is poorly managed. Although improvements in pain management in the hospital setting have been made, pain at home is particularly challenging largely given issues related to adequate assessment. The proposed project will attempt to

refine an existing mHealth pain sensing system to be relevant to cancer pain experienced in the home setting with a focus on the patient-caregiver dyad experience.

Investigator/research team: The PI and team have the requisite experience and training to complete the proposed project.

Approach: The PI and team have worked to move this study forward and have collected pilot data to accomplish aim 1 of the first resubmission, which included data on experience of cancer pain in the home setting and feedback on BESI-C prototypes as well as variables in include within the home setting. These data support the significant of pain in adults with cancer in the home setting and provide positive feedback on the BESI-C system as well as support for the variables included in the system.

Accordingly, the team has revised the protocol to increase the sample size for the pilot assessment of the BESI-C, include time for optimization of the system using an iterative approach, and the addition of an exploratory aim (2) that aims to explore methods for sharing data collected by the BESI-C among stakeholders. This is an important aim as the clinical utility of such a system is key and it will be important to identify how the system will impact clinical management of pain.

The PI and team have worked to address concerns with data protection and concerns regarding cost and dissemination potential of the system. It is clear that the PI has worked to adequately address concerns raised; the only lasting concern of this reviewer is the potential for this system to have widespread impact given its complexity (cost and set up) and this is a concern that is inherent in this system. It is just not clear that the BESI-C will have the necessary clinical relevance to impact pain management protocols.

Environment: The environment will contribute to the success of this project.

PART III BUDGET AND JUSTIFICATION OF BUDGET:

In general, the budget appears appropriate to the project. There is some lack of clarity in the travel budget as the individual amounts do not equal the total requested costs.

PART IV USE OF HUMAN OR ANIMAL SUBJECTS:

Appropriately addressed.

PART V OVERALL RECOMMENDATIONS:

Dr. LeBaron has proposed an outstanding project that addresses a significant issue in patients with cancer in the home setting. Incorporating an existing mHealth assessment program that will involve stakeholders to tailor the program to the experience of cancer pain is an innovative means of addressing the challenges of assessment of pain at home in patients with cancer. It is not clear that this system will have the potential to have widespread clinical impact in the management of cancer pain but given the pilot nature of this study/funding mechanism, it could serve as a foundation to provide support for the utility of the system and inform the use of mHealth in the management of cancer pain at home.
